# Enriching the Value of Patient Experience Feedback: Web-Based Dashboard Development Using Co-design and Heuristic Evaluation

**DOI:** 10.2196/27887

**Published:** 2022-02-03

**Authors:** Mustafa Khanbhai, Joshua Symons, Kelsey Flott, Stephanie Harrison-White, Jamie Spofforth, Robert Klaber, David Manton, Ara Darzi, Erik Mayer

**Affiliations:** 1 Patient Safety Translational Research Centre Imperial College London National Institute for Health Research/Institute of Global Health Innovation London United Kingdom; 2 Imperial College Healthcare NHS Trust London United Kingdom

**Keywords:** patient experience, friends and family test, quality dashboard, co-design, heuristic evaluation, usability

## Abstract

**Background:**

There is an abundance of patient experience data held within health care organizations, but stakeholders and staff are often unable to use the output in a meaningful and timely way to improve care delivery. Dashboards, which use visualized data to summarize key patient experience feedback, have the potential to address these issues.

**Objective:**

The aim of this study is to develop a patient experience dashboard with an emphasis on Friends and Family Test (FFT) reporting, as per the national policy drive.

**Methods:**

A 2-stage approach was used—participatory co-design involving 20 co-designers to develop a dashboard prototype, followed by iterative dashboard testing. Language analysis was performed on free-text patient experience data from the FFT, and the themes and sentiments generated were used to populate the dashboard with associated FFT metrics. Heuristic evaluation and usability testing were conducted to refine the dashboard and assess user satisfaction using the system usability score.

**Results:**

The qualitative analysis from the co-design process informed the development of the dashboard prototype with key dashboard requirements and a significant preference for bubble chart display. The heuristic evaluation revealed that most cumulative scores had no usability problems (18/20, 90%), had cosmetic problems only (7/20, 35%), or had minor usability problems (5/20, 25%). The mean System Usability Scale score was 89.7 (SD 7.9), suggesting an excellent rating.

**Conclusions:**

The growing capacity to collect and process patient experience data suggests that data visualization will be increasingly important in turning feedback into improvements to care. Through heuristic usability, we demonstrated that very large FFT data can be presented in a thematically driven, simple visual display without the loss of the nuances and still allow for the exploration of the original free-text comments. This study establishes guidance for optimizing the design of patient experience dashboards that health care providers find meaningful, which in turn drives patient-centered care.

## Introduction

### Patient Experience

Understanding patients’ experience of health care is central to the process of providing care and is a fundamental pillar of health care quality. It is now widely acknowledged that patients want to give feedback about health care [1] and that staff should be listening to what their patients say about the experience of being in the hospital. However, whether staff can use this feedback to make changes to improve patients’ experiences is now a national initiative [2-6]. This pertains to differing areas of the health care system, from senior management at the level of the hospital board down to individual frontline health care staff. There is a concern that the ever-growing collection of feedback is not being used for improvement but rather represents a *tick box mentality* of organizations thinking that they are listening to their patients’ views but not actually doing so [7]. Several studies have looked at teams of frontline staff to understand how ward staff can engage with patient feedback to make meaningful improvements [1,4,5,8]. Most of the literature in this area finds that despite enthusiasm to make improvements and the vast rhetoric around this, proactive changes are often minimal and largely concentrated on “quick fixes” [3].

### Using Patient Experience Data to Drive Change

Health care organizations within the English National Health Service (NHS) have received recent encouragement to understand the ways in which they use patient feedback to improve care [9]. NHS England and NHS Improvement have implemented changes in patient experience data collected via the Friends and Family Test (FFT). One area of focus is placing greater emphasis on the use of FFT data to drive improvement. For health care organizations to act on this policy change, they need to tackle both macrolevel factors (how organizational structures are unwittingly preventing progress) and microlevel factors (how individual clinicians and teams of staff have difficulty engaging with the data sources) [4]. An organizational strategic focus that prioritizes use over collection and ensures data are relayed to staff by patient experience teams in an accessible, straightforward, and engaging manner is required. Staff training on both quantitative and qualitative analytical techniques and quality improvement (QI) methodologies is also needed. There should be an organizational emphasis where patient experience data collected can be meaningfully used by frontline staff.

### Visualizing Patient Experience Data Through Web-Based Dashboards

There is some evidence that implementing quality dashboards provides constant access to information that can improve adherence to quality guidelines and may help improve patient outcomes [10]. Key reports have called for comprehensive, real-time health care information technology to be integrated into clinical and management processes in health care to improve quality and patient safety [11-13]. A recent report by the National Institute for Health Research [14] recommends that health care organizations produce dashboards and describes dashboards as essential tools to help staff understand areas for improvement in a timelier manner. Visualization of patient feedback is crucial for helping frontline staff and key stakeholders make sense of the structure and underlying patterns in their patients’ experiences. The insights gained from these underlying patterns have the potential to answer vital questions at the point of care [15]. To facilitate this, engaging staff and patients using a co-design approach to visualize feedback is likely to result in sustainable improvements at a local level. Co-design is a process in which targeted end users and other relevant stakeholders form a partnership with researchers and work together on all aspects of intervention development, from needs assessment to content development, pilot-testing, and dissemination [16]. Co-designed interventions may be more effective than traditional approaches where interventions are largely designed by researchers and clinicians. This approach increases the involvement of key stakeholders by encouraging a bottom-up approach, thereby helping health care organizations think differently [17]. The aim of this study is to develop a patient experience dashboard with an emphasis on FFT reporting, as per the national policy drive. An iterative process involving co-design with key stakeholders was used to develop the dashboard, followed by heuristic usability testing.

## Methods

### Setting

This study was conducted at a large London NHS trust. Alongside other health and care partners, the trust caters to a population of approximately 2.3 million people across its 5 sites. Services include accident and emergency, inpatient, outpatient, and maternity, which routinely collect FFT patient experience data. At least one stakeholder from each of the 4 service settings participated in this study.

### Study Design

**Figure 1:**
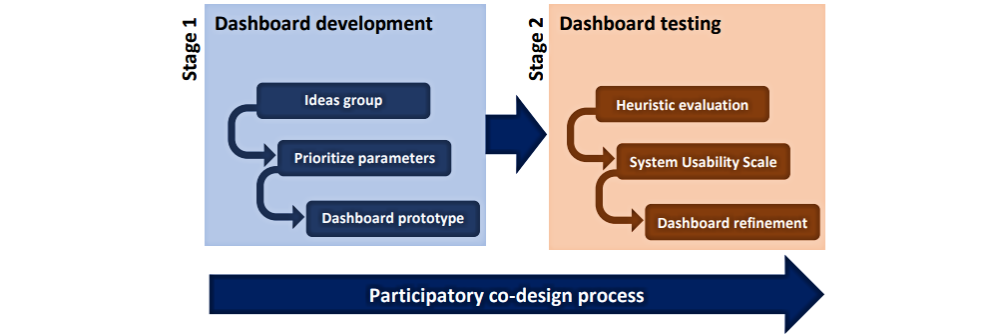
Participatory co-design process used in the study, including stage 1 (developing the dashboard) and stage 2 (testing the dashboard).

This study had two key design stages: (1) dashboard development and (2) dashboard testing (Figure 1). Stage 1 followed a participatory co-design process, which involved key stakeholders (namely health care staff, managers, and a patient representative) in the design of the dashboard. Stage 2 involved heuristic assessment to conduct an informal usability inspection of the dashboard to evaluate whether the user interface of a system adhered to a set of usability principles known as heuristics [18,19]. An invitation letter and a participant information sheet were emailed to the key stakeholders. Informed consent was obtained before interview participation. The researchers led the ideas groups, facilitated and summarized the discussions, took field notes, and made audio recordings. The study received ethical approval from North East–Tyne and Wear South Research Ethics Committee, 17/NE/0306, and took place between April 2018 and February 2019.

### Patient Experience Reports

Retrospective FFT data were used for the intervention from January 2017 to July 2017. We extracted approximately 69,285 FFT responses and associated comments, which were considered adequate to demonstrate the satisfactory accuracy of the text analytics software [20]. Free-text fields identifying favorable service (*What did we do well?*) and areas requiring improvement (*What could we do better?*) were extracted from the patient experience reports across 4 care settings. Using language analysis, free-text comments were themed according to the 2011 English NHS Patient Experience Framework [21] (Textbox 1). The framework was developed by the NHS National Quality Board to guide the measurement of patient experience across the NHS. The framework outlines the elements that are critical to the patients’ experience of NHS services. Sentiment analysis was performed for each free-text comment (ie, positive, negative, or equivocal; Textbox 2). The free-text data (themes and sentiments) from the language analysis and associated FFT parameters were presented to the key stakeholders to develop a bespoke dashboard.

**The eight themes that outline the elements that are critical to patients’ experiences [21]. The themes in italics were added by the research team to include comments that did not fall into the original framework themes.**

**Patient Experience Framework themes**

- Respect for patient-centered values, preferences, and expressed needs
- Coordination and integration of care
- Information, communication, and education
- Physical comfort
- Emotional support
- Welcoming the involvement of family and friends
- Transition and continuity
- Access to care
- Staff
- General
- Unclassified

**The four sentiment categories used to classify patient experience Friends and Family Test free-text comments.**

**Sentiment categories**

- Positive
- Neutral
- Negative
- Unclassified

### Participatory Co-design

In the co-design process, the concepts *Ideas groups*, *Stakeholder needs*, and *Prototyping* were used as described in the Health Service Co-design toolkit [22]. This meant having an iterative refinement process that was reactive to the participants’ feedback. Ideas groups are a tool used to brainstorm ideas for improvement and ways of implementing them in clinical practice. A stakeholder needs table was a useful tool for sketching out possible improvements near the start of the co-design as well as deciding on key areas for improvement and specific improvements later. Prototyping was used to test new products to see if they worked and was a useful way to engage and stimulate creativity among the stakeholders taking part in ideas groups [22].

#### User Needs for Patient Experience (FFT) Reporting

Through purposive sampling, we began by identifying staff to act as co-designers within the patient experience team, followed by lead nurses and junior staff based in 4 care settings: inpatient, outpatient, accident and emergency, and maternity. This strategy helped ensure that we included staff that were either directly or indirectly involved in patient care. A criterion required the interviewees to have a good overview of patient experience feedback, including the FFT, and be currently using all or part of this service in their everyday activities.

Stakeholders came together in ideas groups, and the aim was described: to develop a dashboard that would allow for succinct visualization of the analyzed free text from the FFT reports to identify areas of improvement in a timely manner. In the first ideas group, we discussed the parameters from the FFT reports, including the free-text language analysis output ideas (Textbox 3) and key requirements of the dashboard that were deemed important. In total, 2 research facilitators (MK and SHW) began each 90-minute focus group with a brief presentation of the language analysis toolkit followed by a display of all the FFT parameters, including the free-text language analysis output, separated by theme and sentiment. The participants then broke into 5 groups of 4 to brainstorm ideas on how the FFT reports (ie, core parameters and key requirements that needed to be incorporated into the dashboard) and the various display formats were sketched, and the reasons were presented by a representative in each group. Each participant then had to independently rank their preferred display format (ie, rank order from 1=*first* to 4=*last*). The interviews were transcribed verbatim and double-checked for inaccuracies. To aid trustworthiness of data collection, the first author checked accuracy against interview audio recordings, and the participants were asked to review the transcript of their interview and any sensitive comments were redacted before analysis.

**Friends and Family Test (FFT) questions, including supplementary questions and associated parameters that are routinely collected as part of the FFT survey.**

**FFT questions**

- How likely are you to recommend our service to friends and family if they needed similar care or treatment?
  Extremely likely
  Likely
  Neither likely nor unlikely
  Unlikely
  Extremely unlikely
  Don’t know
- What did we do well?
  Patient experience theme
  Sentiment
- What could we do better?
  Patient experience theme
  Sentiment

**Associated parameters routinely collected as part of the FFT survey**

- Date
- Hospital
- Division
- Ward or clinic
- Language used
- Channel
- Responder (patient, carer, or family)
- Gender
- Ethnicity
- Age range
- Disability

#### Development of the Prototype Dashboard

A prototype was developed and sent out to all the participants. Within 2 weeks, a member of the research team (MK) visited each participant to understand stakeholder needs and gather information on the prototype design (including layout, colors, and information presented on the dashboard) and suggestions for improvements. In general, these feedback sessions lasted approximately 10 to 15 minutes.

### Heuristic Evaluation and Usability Testing

The primary goal of this evaluation was to reduce errors in interpretation and accommodate rapid comprehension, which is critical for using FFT reports in a timely manner. This heuristic evaluation was our initial step toward the development of FFT-visualization-specific heuristics. For this study, we used a validated heuristic evaluation checklist developed to evaluate systems that produce information visualizations [23]. The principles from the heuristics by Nielsen [24] were combined with heuristic principles developed specifically to evaluate information visualization. The use of evaluators who are experts in visual design and understand the analytic intent of the visualizations was important. This was conducted by JS, who has health and design expertise, and by RK and MU, who have health, design, and QI expertise. The checklist consists of 10 usability principles substantiated with 49 usability factors. If the factor was present, the evaluator gave a score of 1 (*Yes*) and, if it was not present, they gave a score of 0 (*No* or *N/A*) [25]. The evaluators drew from heuristic principles related to visual and graphical perception and best practices in graph design as well as years of experience in clinical practice and QI.

The System Usability Scale (SUS) [26], which is a validated posttest questionnaire, was used to measure user satisfaction with product usability. It consists of 10 statements that are scored on a 5-point scale of strength of agreement that captures ratings of electronic devices or systems, including respondent assessments of future use, complexity, ease of use, and perceived usefulness of the display of results. The questionnaire provides a score (range 0-100) based on a participant’s rating of 10 statements regarding a product’s usability. Higher scores indicate greater satisfaction with usability. As a general rule, a system with a score of >70 has acceptable usability; a lower score means that the system needs more scrutiny and continued improvement [27].

### Data Analysis

Data from the ideas groups and from the open-ended questions in the questionnaires were evaluated, discussed, and summarized by the research group. As the aim was to identify improvement ideas expressed by the participants and evaluate the intervention, the data were summarized without an in-depth qualitative analysis. Descriptive statistics were used to describe the participants’ background characteristics. Frequencies and proportions were used to describe the outcomes of the questionnaires and were calculated using Microsoft Excel (version 2019).

The SUS was scored by converting responses to a 0-4 scale (4 was the most positive response). The converted responses were added and multiplied by 2.5, as per the scoring instructions, giving a range of possible values from 0 to 100. Descriptive statistics were used to summarize the SUS scores across all the evaluators of the system. The output from a heuristic evaluation is a summary list of usability problems identified by the group of evaluators. The scores for each heuristic were calculated by dividing the total number of factors (points) awarded by the total number available. The higher the score, the more usable the system was considered to be.

## Results

### Co-designers’ Characteristics

A total of 20 co-designers were recruited for this project (Table 1). We selected co-designers with a variety of characteristics in terms of their professional background, the service settings (division) they were employed in, and whether they were clinical or nonclinical to ensure that the development of the dashboard took into account a diversity of participants.

**Table 1:** Characteristics of the co-designers (N=20).

Characteristic					Participants, n (%)
**Professional background**					
	Nursing and midwifery			6 (30)	
	Allied health			2 (10)	
	Medical			2 (10)	
	**Nonclinical service**				
		Patient experience	3 (15)		
		Quality improvement	3 (15)		
		Data analytics	2 (10)		
		Health care design	2 (10)		
**Division**					
	Surgery and cancer			3 (15)	
	Medicine and integrated care			4 (20)	
	Women and children, and clinical support			3 (15)	
	**Nonclinical service**				
		Patient experience	3 (15)		
		Quality improvement	3 (15)		
		Data analytics	2 (10)		
		Health care design	2 (10)		

### Participatory Co-design Process

The participants were generally enthusiastic about the development of a visualization tool for displaying FFT data and, in particular, the free-text comments in a meaningful way and in near real time. Most felt that a dashboard might highlight areas that required improvement as well as areas that had been improved, which might enhance how staff interacted with FFT data. Results from the ideas group were separated into the FFT parameters that were deemed important, key requirements that should be considered during development, and ranking of the 4 dashboard sketches.

#### FFT Parameters

The feedback from the ideas group highlighted that, although all parameters were important, only a select number were chosen to be displayed on the opening screen, whereas the rest could be accessed through a tab. The most important parameters were date, ward or division, sentiment, and patient experience theme. As the FFT is anonymous, most staff thought that segmenting the feedback by demographics had a risk of identifying the patient, especially if the reports were accessed in real time. The date of feedback was crucial to respond in near real time and to look for trends and assess progress over time. The ward or division was required so that feedback could be accessed by all staff, ensuring transparency as well as identifying opportunities for improvement (eg, from other wards with similar specialties or patient profiles). The FFT score was considered less insightful in understanding where improvements needed to be made, and the participants unanimously agreed that the free-text option should take precedence when displaying the FFT data and should be displayed on the opening dashboard screen. Individual sentiment was not considered useful as most were positive; however, the average sentiment of each patient experience theme was the preferred approach. The participants highlighted that negative comments could be sandwiched between positive comments and vice versa and that staff felt it was important to consider this context rather than separate the positive comments from the negative comments. Therefore, the themes with average sentiment were displayed as *to improve* in relation to the question *What could we do better* and as *doing well* in relation to the question *What did we do well?* Despite the free-text comments being clustered into themes, frontline staff agreed that they should have the opportunity to drill down into specific or unusual comments for further manual analysis to gain additional insight.

#### Key Dashboard Requirements

We summarized feedback from the ideas group on what an ideal dashboard would require in relation to FFT reporting (Textbox 4). The statements reported related to accessing the reports in an easy and understandable manner that allowed staff to assimilate the pertinent information in a short time frame, thereby addressing patients’ experiences as they are reported.

**A summary of the key requirements for the dashboard from the ideas group.**

**Key requirements for the dashboard**

- Easy access to the data in a visual and usable format
- Data provided in a way that can be engaged with by frontline staff
- Summary data that can be mined down to individual comments
- Locally relevant information displayed for comparison across similar wards
- Ability to see change through the months or years
- Facilitating discussion with the executive board acting as leverage to drive change
- Information provision in near real time
- Positive feedback, celebratory sharing with teams
- Free text better than scores
- Giving all ward staff ownership of the data, narrowing the
  skill gap
- Content should not be overwhelming
- Imparting a positive mindset to improvement as
  core activity

#### Dashboard Design Popularity

A total of 4 main dashboard design formats were presented by the 4 groups: bar chart, line graph, bubble chart, and pictograph. Table 2 shows the preference rankings. The bubble chart was ranked first, being the most preferred by the participants (*P*<.001). This was primarily because the participants favored displaying the experience visualizations using the same format as other visualizations currently used in the organization, for example, the Patient Safety dashboard. This consists of the safety incidents using a bubble chart, which is currently used by all staff within the organization.

**Table 2:** Mean preference ranking (1=lowest and 4=highest) for each display dashboard among the co-design participants (N=20).

Dashboard design format	Preference ranking, mean (SD)
Bar chart	3 (0.86)
Line graph	1.35 (0.59)
Bubble chart	3.5 (0.69)
Pictograph	2.05 (0.89)

#### Development of the Dashboard Prototype

**Figure 2:**
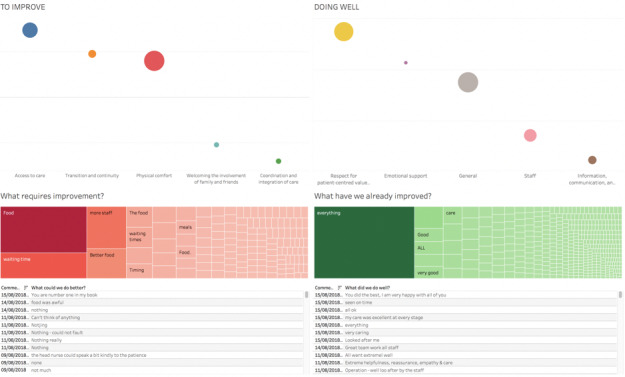
Prototype dashboard presented in a bubble chart, where inpatient free-text comments are split by the top 5 themes and sentiment (negative [to improve] on the left and positive [doing well] on the right). A word heat map demonstrates the most common words found within the free-text comments, followed by individual comments.

**Figure 3:**
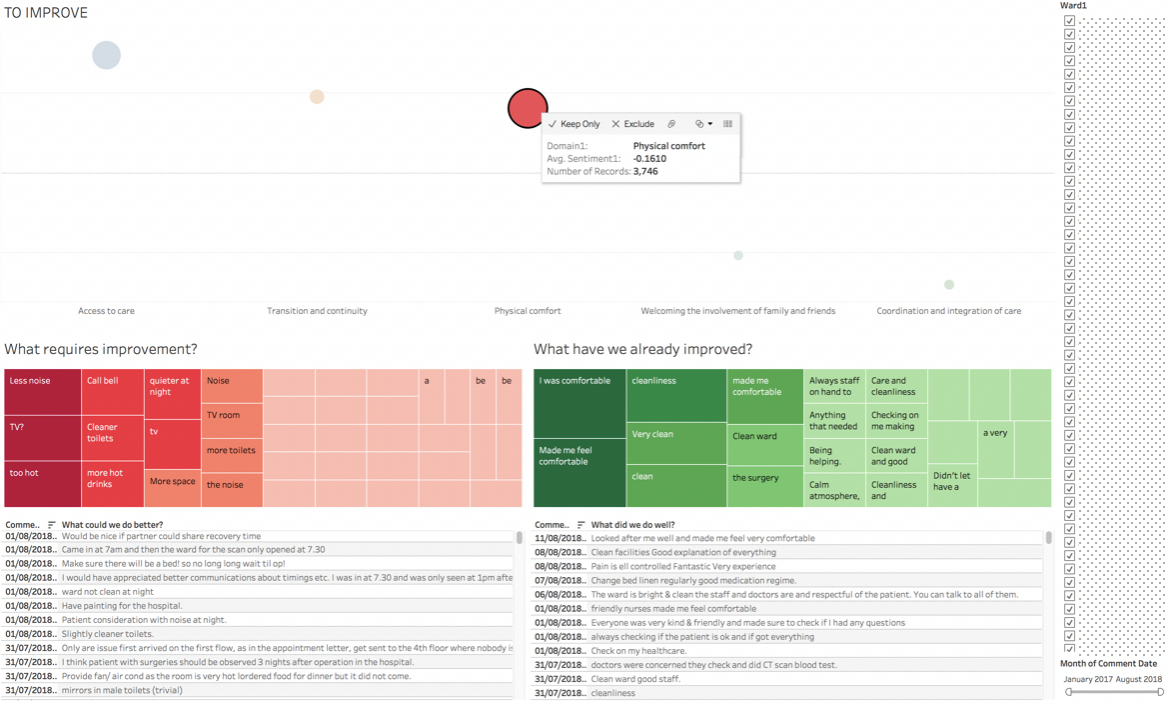
This display demonstrates only negative (to improve) inpatient comments with web-based features. The word heat map shows the most common comments split by negative sentiment in red and positive sentiment in green, followed by individual comments that describe physical comfort only.

On the basis of observations, interviews, and feedback, we developed an information-rich suite of display implemented in Tableau (Tableau Software) that provided at-a-glance information of FFT-reported free-text data. Tabs for each dashboard were visible across all views that document the individual steps taken to develop the final dashboard. However, for the dashboard testing, the dashboard was presented on a Tableau reader, which does not allow the user to make any changes. The census overview was the opening screen, which contained the top 5 themes with the most negative sentiment presented on the left as *to improve* and the top 5 themes with positive sentiment presented on the right as *doing well* for all inpatient comments (Figure 2). A traffic light color coding system was developed (ie, the most negative sentiment was coded as red and the most positive sentiment was coded as green presented as a word heat map; Figure 2). The user had the ability to configure their preferences by isolating the visualization based on positive or negative sentiment instead of side-by-side comparison (Figure 3). There was a date range toggle bar and a list of wards on the right side of the dashboard screen that could be selected by the user or where the number of comments and average sentiment in each theme *bubble* could be viewed by hovering over each data point. The final version embodies a dashboard where users can interact with the visualization, use filters to modify the display, and select an individual theme *bubble* that presents all the free-text comments within that specific theme (Figure 3).

### Heuristic Evaluation and Usability Testing

Most cumulative scores from the 3 participants who took part in the heuristic evaluation had no usability problems (18/20, 90%), had cosmetic problems only (7/20, 35%), or had minor usability problems (5/20, 25%). The areas requiring attention recorded by a higher severity rating were user control and freedom, and consistency and standards. The percentage score was lowest in user control and function (60%) followed by consistency and standards (66.7%), and the highest score was flexibility and efficiency of use (90%) followed by visibility of system status (88.3%; Table 3). The heuristic evaluators also made suggestions for their implementation. Specific issues that required addressing were having the dashboard service settings consistent (eg, inpatient compared with maternity; minor usability problem), making the data accessible on hovering the mouse (cosmetic problem only), ensuring the data were presented as the 2 supplementary questions (cosmetic problem only), changing all font to *Arial* (cosmetic problem only), increasing the size of the *bubble* (cosmetic problem only), presenting the data in descending order and having the month toggle bar at the top of the screen (minor usability problem), and excluding the comments themed as general (minor usability problem). There was unanimous feedback that the word-based heat map, although useful, did not add much to gaining knowledge and made the dashboard cluttered (minor usability problem); however, the color coding should remain for the headings (ie, green for positive sentiment and red for negative sentiment) and the caption above the comments should be removed (cosmetic problem only). As the dashboard was presented on a Tableau reader that did not allow the participants to make any changes, some of the questions about user control and freedom did not apply; however, the free-text responses from the participants were taken into account.

**Figure 4:**
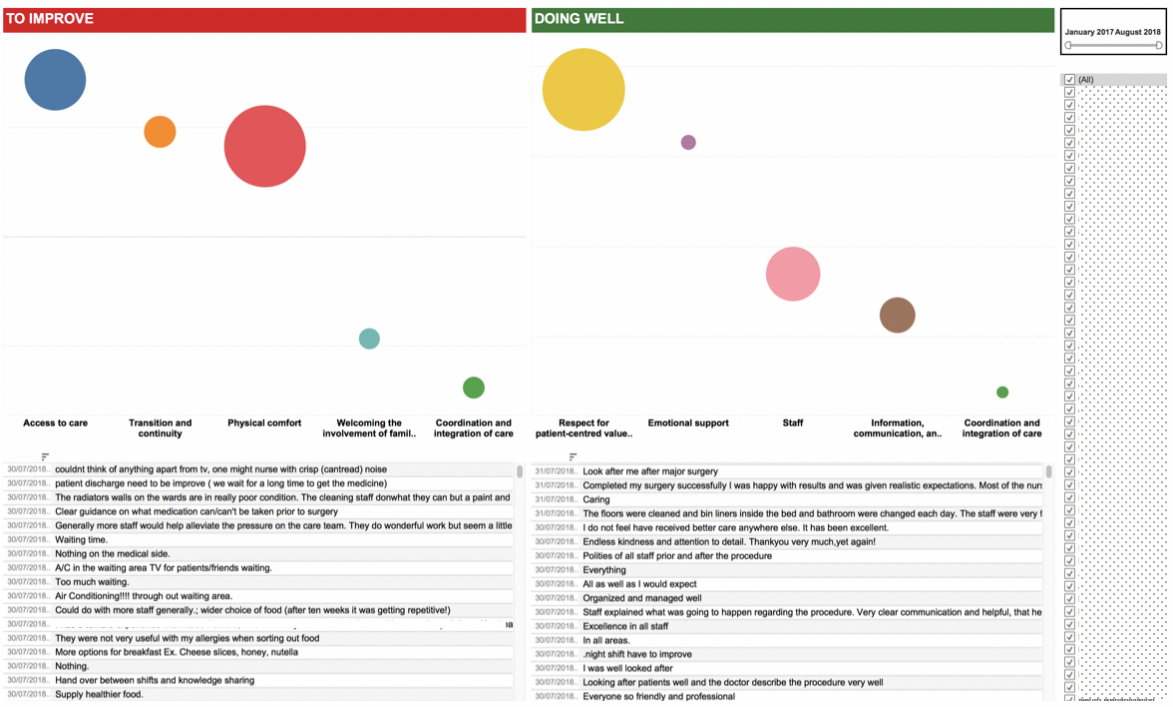
Final dashboard amended following heuristic evaluation, which was tested using the System Usability Scale. This dashboard presents inpatient comments divided into the top 5 themes in descending order with negative (to improve) and positive (doing well) sentiment.

Amendments were made accordingly, and the final dashboard (Figure 4) was tested on the participants for satisfaction. The mean SUS score was 86.97 (SD 5.79), and the median score was 87.5. Participants from a nursing background and those from the patient experience team with a nonclinical background had the highest scores.

**Table 3:** Mean heuristic evaluation ratings for the prototype dashboard.^a^

Heuristic evaluation (maximum score)	Overall severity rating, mean (SD)	Score, mean (SD)	Score result (%)
Visibility of system status (6)	0 (0)	5 (1)	83.3
Match between system and the real world (5)	0.7 (1.2)	4 (1)	80
User control and freedom (5)	1.7 (0.6)	3 (1)	60
Consistency and standards (6)	1.3 (0.6)	4 (1)	66.7
Recognition rather than recall (4)	0.7 (1.2)	3 (1)	75
Flexibility and efficiency of use (7)	0.7 (0.6)	6.3 (0.6)	90
Esthetic and minimalist design (7)	0 (0)	5 (1)	71.4
Spatial organization (3)	0 (0)	2.3 (0.6)	77.8
Information coding (2)	0.7 (0.6)	1.3 (0.6)	66.7
Orientation (4)	0 (0)	3 (0)	75

^a^The maximum score that each question can receive is shown in parentheses. The overall severity rating score ranges from 0 (no usability problem) to 4 (usability catastrophe), and the mean overall severity rating is shown. The score result is calculated as a percentage of the maximum score.

## Discussion

### Principal Findings

Quality dashboards integrating health care data offer innovative means of providing metrics that can facilitate QI [28]. We demonstrated an iterative approach to developing a web-based dashboard using free-text FFT data collected as part of a national drive to improve quality. To the best of our knowledge, this is the first study using co-design principles and heuristic evaluation to develop a dashboard to visualize free-text FFT data for QI.

The literature suggests that data availability is a crucial precondition for the development of dashboards [29]. However, this presents health care organizations with a challenge as data are often presented in quantitative and summative format, whereas staff also desire qualitative information [29,30], confirming our findings. Therefore, facilitated by the findings from our co-design study, we extended the scope of the FFT data by augmenting the dashboard with associated free-text data, which not only provides a richer narrative but also makes the data more meaningful to staff [5].

Previous research shows that actual dashboard development often starts with the translation of available data into useful dashboard content [29], and the use of focus groups facilitates a better understanding of the needs and wishes of the stakeholders when formulating a design [28]. Our focus groups were guided by co-design principles—involving stakeholders in the design and development of visualization tools increases functionality and usability by meeting stakeholder requirements, thereby improving the quality of the system and increasing the likelihood of achieving intended health outcomes [31]. The literature on dashboard development mostly addresses the technical aspects of development processes while overlooking the organizational aspects [28]. Combining technical and organizational aspects into one comprehensive development process was vital for addressing this challenge. Our 2-stage approach illustrates a range of stakeholder engagement methods, dashboard prototypes, and design insights on meaningful dashboard content, format, and clinical use.

A stark finding from Weggelaar-Jansen et al [28] revealed that no hospital taught health care professionals or managers to understand statistical measurements and the related graphics to help them understand the dashboard. In addition, the studies [32,33] that used co-design to create a dashboard for Cancer Patient Experience Survey data and the patient experience toolkit did not clearly address the time poverty that is a growing challenge, hindering health care staff from having dedicated time within their duties to engage with the dashboard. Addressing these issues was a key requirement that was raised during the ideas group discussion in our study. The participants raised the issue that the pre-existing format of patient experience reporting used too much technical language, which required training in data analysis and statistics to facilitate its full understanding to then use the results appropriately. This aspect was particularly important to ensure that the dashboard was interwoven into the daily activities of frontline staff. Therefore, the participants unanimously agreed to create a patient experience dashboard that would follow an existing format that was established and widely used in the organization—the Patient Safety dashboard. This meant that the prototype evolved and adapted exploring similarities with the Patient Safety dashboard but displayed patient experience data that would enable staff to meaningfully engage with the new dashboard without *costing* so much of their time. Adopting a new visualization that was different from the format of the currently used Patient Safety dashboard would have resulted in a steep learning curve and possibly discouraged and disengaged staff, thereby failing to translate FFT reports into actionable interventions.

To achieve a broadly comprehensible layout, we ensured that the real-time graphic and visual presentation of the content fit the purpose of the dashboard [10]. Previous studies [32,33] have highlighted the use of visual and physical media as a form of sharing and communicating, which helped remove barriers to mutual understanding. Short summaries (eg, dashboards and graphs) are essential tools to help staff understand areas for improvements quickly [32] as the presentation of data enables them to navigate it in ways that answer questions specific to their service or to particular patients [33]. Embedding the outcomes of the participatory co-design process informed the development of the prototype, and validation with stakeholders using established usability techniques provided reassurance that the approach had value for staff.

To enable stakeholders to customize dashboard content to their own needs, research suggests that health care organizations add 3 main functionalities, namely, drill-down, filter, and alert functions [28]. Our dashboard fulfilled these criteria with the availability of filters to modify the display and select an individual theme *bubble* to present all the free-text comments within that specific theme and sentiment, and the ability to view the 5 most important themes as determined by sentiment. An interesting trade-off was observed between the need for detail and the need for brevity during the usability evaluation. Feedback from the heuristic evaluation demonstrated that the appearance of the dashboard needed to be simple and that it should not look like a major task to understand the features. Through a series of adaptations, we addressed the cosmetic problems (n=7) and minor usability problems (n=5) to deliver a punchy dashboard and still contain all the desired features and requirements that had been highlighted during the co-design process. These dashboard features specifically improved staff engagement and empowerment by attracting their attention and stimulating them to pay attention to the information of interest, keeping their attention and interest for longer periods, and providing a greater depth of content [19]. This meant that the final dashboard was ultimately designed for use by all health care staff, as demonstrated by the usability score. In general, it is considered that usable products should have SUS scores of >70; our prototype had a mean SUS score of 86.97 (SD 5.79), suggesting acceptable usability [27]. The highest scores came from the participants from a nursing background and the patient experience team, which is an encouraging result. We hope that this translates to sustained engagement in the use of the dashboard and, as a consequence, generates a body of *patient experience ambassadors* to help raise awareness of the use and importance of patient experience dashboards across the organization.

### Limitations

This study was conducted at a single hospital, and all participants were employees within the hospital, thereby causing selection bias. Although this is a limitation, the principles underlying the development of the dashboard are transferable across different hospitals that collect patient experience feedback. This dashboard was only accessible to participants in the study; therefore, usability was evaluated on the same participants from the co-design process, inviting reporting bias on the final SUS score. Another important limitation that has implications beyond this particular co-design study is the potential for the idealization of the work context by the staff involved. When staff are taken off the ward and given some time and space to be involved in co-designing an intervention, which later they will deliver in a busy ward, they are not necessarily able to anticipate the difficulties that they will face. Alternatively, they may ignore these challenges because they are fearful of admitting to them in a group setting, particularly in a group that includes patients.

It could be said that we are currently at a key pivotal moment in terms of the patient experience debate in relation to both national and local policy and what is occurring *on the ground*. This is because there is an ever-clearer and acknowledged push for improvement to arise from patient feedback, but individuals and systems are constrained from doing so. This study has attempted to address the point in the National Institute for Health Research report [14] that there is still uncertainty as to how to present patient experience data in a meaningful and granular way that stimulates local action. An important result and advantage of our study’s approach is that it draws together very large FFT data into a thematically driven, simple visual display without loss of the nuances that other manually based methods can have, and it can still allow for exploration of the original free-text comments.

### Conclusions

The use of visualization techniques such as dashboards is increasing in response to staff needs for summarized, easily interpreted patient information at the point of care. In this study, through a participatory co-design process and usability heuristic evaluation, we developed and refined a dashboard displaying patient experience, namely FFT data, for use by staff and key stakeholders in near real time. The contributions of this study establish guidance for optimizing the design of FFT dashboards that key stakeholders, especially frontline staff, find meaningful and, in turn, support patient-centered care. The impact of this work is being measured in an ongoing trial, the results of which will guide future refinement, integration with electronic health care records, and steps toward dissemination.

## Abbreviations

FFT: Friends and Family Test
iCARE: Imperial Clinical Analytics Research and Evaluation
NHS: National Health Service
QI: quality improvement
SUS: System Usability Scale
